# Leptin Receptor Gene Polymorphism and the Risk of Cardiovascular Disease: A Systemic Review and Meta-Analysis

**DOI:** 10.3390/ijerph14040375

**Published:** 2017-04-03

**Authors:** Lei Wu, Dali Sun

**Affiliations:** 1 Department of Epidemiology, Institute of Geriatrics, Chinese People‘s Liberation Army General Hospital, Beijing 100853, China; 2 Department of Nanomedicine, Houston Methodist Research Institute, Houston, TX 77072, USA; samio4762@gmail.com

**Keywords:** Leptin receptor gene polymorphism, cardiovascular disease, meta-analysis, Lys109Arg, rs6700896

## Abstract

*Objective*: Few studies have assessed the association between leptin receptor (LEPR) gene polymorphism and the risk of cardiovascular disease (CVD). Of the few epidemiological studies on this topic, the results are still controversial. *Methods*: PubMed and Embase were screened for studies from their inception to 9 October 2016. The pooled odds ratio (OR) with the corresponding confidence intervals (CI) were used to measure the effect size for studies that reported the association under allelic, homozygous, and dominant models. Pre-specified characteristics were conducted in the subgroup analysis. Heterogeneity between subgroups was evaluated by meta-regression analysis. *Results*: Seven eligible studies involving 44,133 participants were included in our meta-analysis. Borderline significant association was observed between the LEPR gene polymorphism (rs1137101, rs1137100, rs6700896, and rs8179183) and the increased risk of CVD with considerable heterogeneity under the allelic model, and the overall pooled OR (95% CI) was 1.10 (0.99, 1.22). The LEPR gene variant rs6700896, 109G allele, and 109GG genotype were significantly associated with the increased risk of CVD. Furthermore, stratified group analysis revealed that the association was more pronounced for stroke. Race-differences might also cause the considerable heterogeneity and non-significant association. *Conclusions*: This is the first systematic review and meta-analysis to investigate the association between LEPR gene variants and CVD risk. Some LEPR gene variants were significantly associated with the increased risk of CVD. However, the present study is limited in its small number of included studies, considerable heterogeneity, and observational study design. Further research is still warranted to confirm the magnitude of the association.

## 1. Introduction

CVD remains a critical public health issue along with the ageing of the population worldwide [1]. In recent decades, the prevalence of coronary heart disease (CHD) has largely increased in Europe and the United States [1]. It has been widely accepted that the occurrence of CVD arises from interactions between environmental and genetic factors [2,3]. To date, more than 40 loci have been proven to be linked with CVD susceptibility through genome-wide association studies (GWAS) [4].

Leptin has been affirmed to regulate various cardiac and vascular effects, including thrombosis, angiogenesis, and cardiac hypertrophy [5]. Furthermore, it also has an impact on the control of metabolism, immunity, and reproduction. Leptin exerts its physiological action through the Leptin receptor (LEPR), which is located on chromosome 1p31. LEPR is a molecule distributed in various tissues [6], and it can mediate the important impact of leptin as a hormone for whole-body energy homeostasis. 

In previous studies, several single nucleotide polymorphisms (SNPs) of LEPR, such as rs1137101, rs1137100, rs6700896, and rs8179183, have been proven to be associated with a variety of chronic diseases (diabetes mellitus, hypertension, and some cancers) [7,8,9]. However, few studies have assessed the association between LEPR gene polymorphism and the risk of CVD, and the results are still controversial. For instance, Abd El-Aziz et al. reported that the carrier of the Gln223Arg polymorphism Gln/Gln genotype decreased the risk of heart failure [10]. However, other studies reported that the 223GG genotype was associated with a significantly increased risk of chronic heart failure or ischemic stroke [11,12]. We speculated that the sample size of only 200 subjects in the study by Abd El-Aziz et al. limited the interpretation of their findings [10]. In addition, participants with different characteristics may also produce diverse results in different studies.

To the best of our knowledge, no evidence from quantitative analysis has evaluated the association of LEPR gene variation and the risk of CVD. Therefore, we conducted a systematic review and meta-analysis analysis to increase the precision of effect estimates, thereby clarifying the relationship between the LEPR gene variant and CVD risk.

## 2. Methods

### 2.1. Literature Search

The present systematic review and meta-analysis was conducted following the guidelines of meta-analysis of observational studies in epidemiology [13]. The PubMed and the Embase databases were searched from their inception to 9 October 2016 for published articles relevant to LEPR gene polymorphism and the risk of CVD. Language restriction was not set in the search process. The detailed search strategy is shown in Supplementary Table S1. In addition, we manually screened the reference lists of related original articles and reviews to identify more potential studies. If multiple publications from the same study were identified, we included the article involving the largest number of subjects. 

### 2.2. Selection Criteria and Data Extraction

Two authors independently searched and selected the publications. Duplicate records were deleted and the titles and the abstracts of all articles were screened. We identified articles as exclusions or further estimations with the following selection criteria: (1) Original studies that reported data relevant to LEPR gene polymorphism and the risk of CVD. (2) Participants aged 18 years or more. (3) Cases were individuals with heart disease (heart failure, coronary heart disease, myocardial infarction, etc.) or stroke (ischemic, hemorrhagic), whereas controls were individuals without these diseases. Disagreements were resolved by discussion. 

Two investigators independently performed the data extraction. We extracted the following data from each eligible article: the first author, the published year, ethnicity, age, and gender of participants, number of cases and controls, method of genetic and outcome measurements, disease type of outcome, allele and genotype frequencies (cases and controls), or the adjusted odds ratios (ORs) or relative risks (RRs) or hazard ratios (HRs) with the corresponding 95% confidence intervals (CIs) of LEPR gene polymorphism and the risk of CVD (the largest number of adjusted confounders). 

### 2.3. Quality Assessment

The Guidelines of Methodological Evaluation of Observational Research (observational studies of risk factors of chronic diseases) [14] and the quality assessment tool of observational studies [15] were used to estimate the quality of each included article. The following five domains were assessed: (1) the design bias, scored 0 to 3 points, (2) selection bias, scored 0 to 4 points, (3) information bias, scored 0 to 5 points, (4) confounding, scored 0 to 3 points, and (5) analysis bias, scored 0 to 2 points. A maximum of 17 points was obtained for the article with the highest quality. Disagreements were resolved by discussion between the two authors.

### 2.4. Statistical Analysis

We used the software Stata, version 12.0 (StataCorp LP, College Station, Texas, TX, USA) and the Review Manager, version 5.2 (The Nordic Cochrane Centre, Copenhagen, Denmark) to perform the statistical analyses. The pooled OR with its corresponding 95% CI was used to measure the effect size for studies that reported the association between LEPR gene polymorphism and CVD risk. Two-sided *p* values of less than 0.05 were considered statistically significant. The pooled ORs (95% CIs) were calculated in three genetic models; allelic model (m vs. M), homozygous model (mm vs. MM), and dominant model (mm vs. Mm + MM). We used the Q test and I^2^ statistic to evaluate the heterogeneity across studies, and I^2^ statistics >50% and/or *p* values < 0.05 was judged as statistically significant. The following pre-specified characteristics were conducted in the subgroup analysis: ethnicity (Caucasian, Asian, mixed), study design (case-control, cohort study), and disease type of cases (heart disease, stroke). Heterogeneity between subgroups was evaluated by meta-regression analysis, and a *p* value of 0.05 or below indicated significant results. By omitting one article at every turn, the influence of a single article on the overall combined results was evaluated in the sensitivity analysis. The possible presence of publication bias was assessed through the Begg and Egger’s tests [16,17].

## 3. Results

### 3.1. Study Identification and Selection

**Figure 1:**
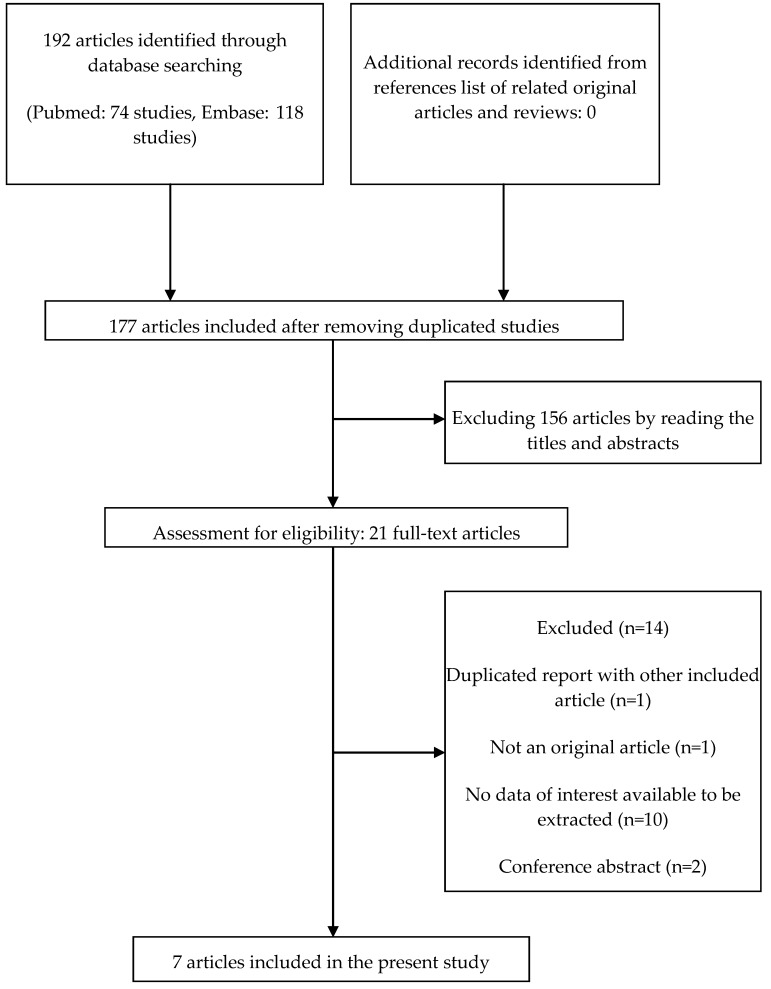
Flow diagram of the articles included in the present systematic review and meta-analysis.

The flow diagram of the articles included in our systematic review and meta-analysis is shown in Figure 1. A total of 192 articles were identified through the initial search process (Pubmed: 74 articles, Embase: 118 articles). After excluding 15 duplicates, 177 articles were included for further assessment. We further excluded 156 articles by reading the title and the abstract of each article, and the remaining 22 full-text articles were assessed for eligibility. Finally, a total of 7 studies were included in the present study [10,11,12,18,19,20,21].

### 3.2. Study Characteristics

Table 1 presents the characteristics of the included studies (two cohort studies, five case-control studies). The published year of these articles ranged between 2009 and 2015. One of the included articles was conducted in Egyptian subjects [10], two studies were performed in Chinese subjects [12,20], three studies were performed in European [18,21] and American subjects [11], and the other one study was performed in both European and Asian subjects [19]. All articles included both male and female participants. The sample size ranged between 200 [10] and 41,275 [19] for a total of 44,133 participants. All the outcome variables were assessed by clinical diagnosis or hospital registration. Six studies reported the outcome of heart disease [10,11,18,19,20,21], and one study reported the risk of ischemic stroke [12]. Four LEPR gene polymorphism loci were included in the present analysis. Five, three, two, and two studies reported the LEPR gene polymorphism of rs1137101 (Gln223Arg) [10,11,12,18,21], rs1137100 (Lys109Arg) [12,18,21], rs6700896 [19,20], and rs8179183 (Lys656Asn) [12,21], respectively. Genotyping of the polymorphisms in LEPR was carried out by the polymerase chain reaction (PCR)-restriction fragment length polymorphism method (RFLP) [10,11,12,18,20,21], or genome-wide association scan [19].

### 3.3. Quality Assessment

The quality assessment of the eligible studies is shown in Supplementary Table S2. The quality score of all studies were all higher than 11 points, with the highest score of 17 points [19]. Common deficits of the study quality were the design bias, selection bias, and confounding. Only two included studies were of cohort design [18,19]. Two studies did not provide information of the eligibility criteria [18,21]. Three studies did not adjust other potential confounders except for age and gender [10,12,20], and one study did not adjust for any confounding variables [21]. The withdraw rate of the two cohort studies was less than 20%.

### 3.4. LEPR Gene Polymorphism and CVD Risk

**Figure 2:**
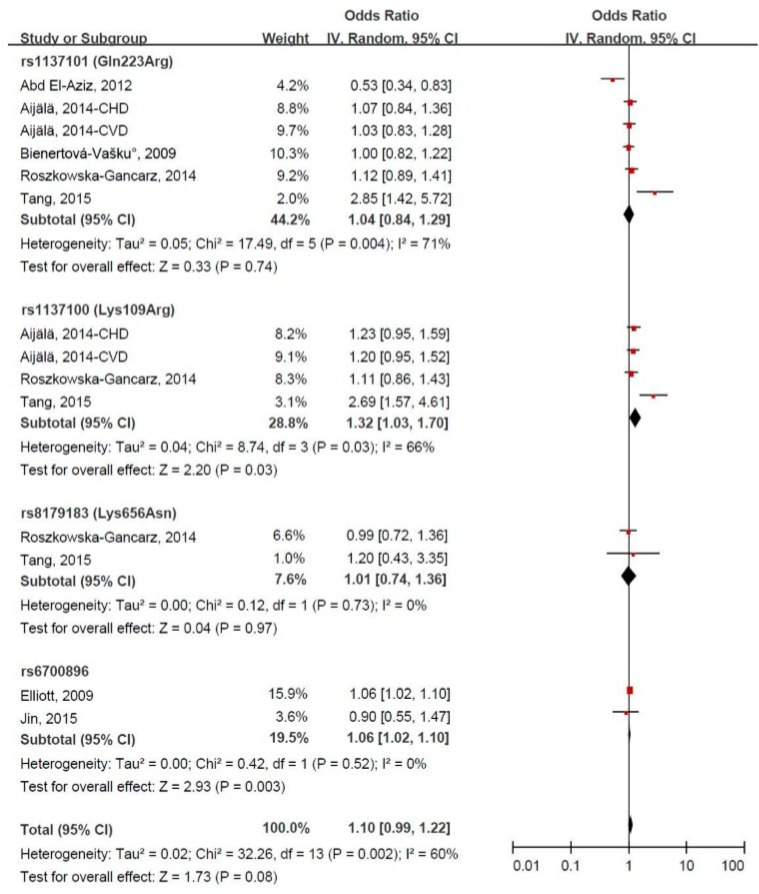
Forest plot of odds ratios (ORs) and 95% confidence intervals (CIs) for the association between leptin receptor gene polymorphism and the risk of cardiovascular disease under the allelic model.

Three different models were used to test the association between the LEPR gene variant and CVD risk. Figure 2 shows the random-effects forest plot for the association between LEPR gene polymorphism and CVD risk under the allelic model. The LEPR gene variant was borderline significantly associated with the increased risk of CVD in 14 comparatives, and the pooled OR (95% CI) was 1.10 (0.99, 1.22), with evidence of significant heterogeneity (I^2 ^= 60%, *p* = 0.002). Both the LEPR gene variant rs1137100 (109G allele) and rs6700896 were significantly associated with the increased risk of CVD, and the pooled ORs (95% CIs) were 1.32 (1.03, 1.70) and 1.06 (1.02, 1.10), respectively. In the sensitivity analysis, exclusion of the study by Abd El-Aziz et al. [10] significantly altered the combined result and decreased the heterogeneity, and the corresponding overall pooled OR (95% CI) was 1.12 (1.02, 1.23). As shown in Supplementary Figure S1a, no publication bias was observed (Egger’s test, *p* = 0.435; Begg’s test, *p* = 0.228). 

**Figure 3:**
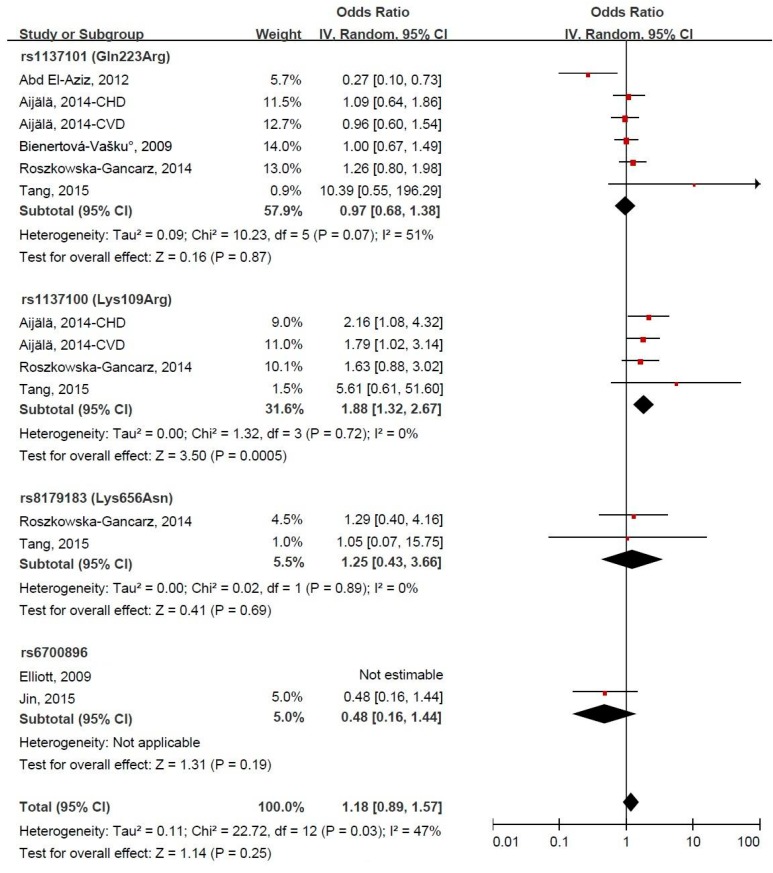
Forest plot of odds ratios (ORs) and 95% confidence intervals (CIs) for the association between leptin receptor gene polymorphism and the risk of cardiovascular disease under the homozygous model.

**Figure 4:**
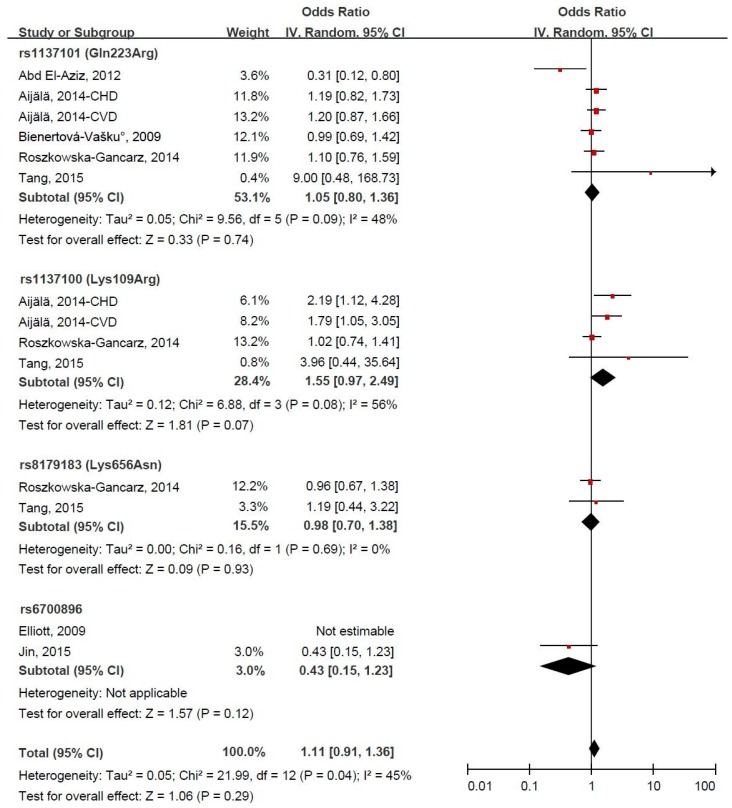
Forest plot of odds ratios (ORs) and 95% confidence intervals (CIs) for the association between leptin receptor gene polymorphism and the risk of cardiovascular disease under the dominant model.

Forest plots for the association between LEPR gene polymorphism and CVD risk under the homozygous model and dominant model are presented in Figure 3 and Figure 4. LEPR gene variants were not significantly associated with CVD risk, and the pooled ORs (95% CIs) were 1.18 (0.89, 1.57) and 1.11 (0.91, 1.36), respectively, both with evidence of significant heterogeneity (*p* = 0.03 and *p* = 0.04, respectively). Under the homozygous model, the LEPR gene variant rs1137100 (109GG genotype) was significantly associated with the increased risk of CVD, and the pooled OR (95% CI) was 1.88 (1.32, 2.67). Exclusion of the study by Abd El-Aziz et al. [10] significantly altered the combined result and decreased the heterogeneity, and the corresponding pooled OR (95% CI) was 1.26 (1.01, 1.58). Under the dominant model, the LEPR gene variant rs1137100 was borderline significantly associated with the increased risk of CVD, and the pooled OR (95% CI) was 1.55 (0.97, 2.49). In the sensitivity analysis, the exclusion of a single study did not significantly alter the combined result, and the pooled ORs (95% CIs) ranged between 1.07 (0.88, 1.30) and 1.15 (0.98, 1.36).

No evidence of publication bias was observed under the homozygous model and the dominant model (Egger’s test: *p* = 0.677, Begg’s test: *p* = 0.428; and Egger’s test: *p* = 0.686, Begg’s test: *p* = 0.428) (Supplementary Figure S1b,c). 

### 3.5. Subgroup Meta-Analysis

Supplementary Table S3 presents that the stratified analyses based on race, and the study design did not significantly affect the association between the LEPR gene polymorphism and CVD risk (*p*-value for difference >0.05 for each group). Subgroup analysis by outcome of disease type significantly affected the association under the allelic model, and the pooled ORs (95% CIs) were 1.06 (0.99, 1.13) for heart disease and 2.42 (1.60, 3.65) for stroke (*p*-value for difference = 0.003).

## 4. Discussion

The present systematic review and meta-analysis identified seven studies involving a total of 44,133 participants. Borderline significant association was observed between the LEPR gene polymorphism (rs1137101, rs1137100, rs6700896, and rs8179183) and the increased risk of CVD with considerable heterogeneity under the allelic model, and the overall pooled OR (95% CI) was 1.10 (0.99, 1.22). The LEPR gene variant rs6700896, 109G allele, and 109GG genotype were significantly associated with the increased risk of CVD. Furthermore, stratified group analysis revealed that the association was more pronounced for stroke. Race-difference might also cause the considerable heterogeneity and non-significant association.

LEPR Gln223Arg is the relatively well-studied polymorphism among all four included SNPs in the present study. The LEPR Gln223Arg G allele and GG genotype have been reported to be linked with some cancers [22,23]. Additionally, the Gln223Arg polymorphism also gives rise to increased susceptibility to Entamoeba histolytica infections in children [24]. However, the findings are conflicting regarding the association with metabolism and altered glucose [25,26]. Our pooled analysis showed that the LEPR Gln223Arg gene variant was not significantly associated with CVD risk. The sensitivity analysis revealed that a study in Egypt significantly altered the combined result [10]. In fact, the frequency of the LEPR 223R allele was highly varied in different ethnicities [27], and the frequency was relatively lower in Egyptians [10]. We speculated that the opposite conclusion between Egyptians and other populations might be due to various genetic backgrounds. In addition, considering the limited sample size (only 100 cases and 100 controls) of the study [10], the probability of a false relationship might have been given. High quality, large sample studies are still warranted to consider the association in different ethnic groups and countries.

Lys109Arg, located in the cytokine receptor homologous 1 (CRH1) domain in LEPR, can affect the function of LEPR and modifies leptin mediated signaling [28,29]. Our results revealed that LEPR Arg109Arg had a protecting effect against the CVD risk. Previous studies have reported that individuals with the LEPR Arg109Arg genotype have lower risks of hypertension, obesity, dyslipidemia, and diabetes [8,30,31,32], which are all important established risk factors of CVD. As a result, these above studies are likely to give mediated evidence to support our study. 

In the present study, we found that the LEPR gene variant rs6700896 was associated with an increased risk of CVD. SNP rs6700896 is a member of the class I cytokine receptor family, which is located in intron 19 of LEPR [33]. It has a role in appetite control, weight regulation, glucose homeostasis, blood pressure regulation, and angiogenesis [30,33]. It is worth noting that all of the above factors are related with CVD. SNP rs6700896 may give rise to the risk of CVD through increasing body weight and adiposity. The significant association was mostly attributed to the study by Elliott et al., which demonstrated the causal association of rs6700896 and coronary heart disease in a very large population from both Asians and Europeans [19]. However, the other study [20] in our pooled analysis did not find a significant result between rs6700896 and coronary artery disease susceptibility. One possible explanation might be the different study population. Additionally, the small sample size of the study by Jin et al. [20] might not enough to observe statistically significant findings.

Although stroke is an important issue and the existing evidence suggests that the association between the LEPR gene variant and stroke may exist, very few studies have evaluated its relationship. Only one study was searched and included in the pooled analysis, and it concluded that the LEPR 109GG and 223GG genotype carriers were associated with a threefold increased risk of ischemic stroke [12]. The threefold effect size is greater than that in other included studies, which assessed the association between the LEPR gene variant and heart disease. The findings revealed that the magnitude of association might be more pronounced for stroke. Further study with a larger sample size is required to prove this finding.

The present systematic review and meta-analysis has several limitations. First, only a small number of studies were relevant to this topic; thus, only seven studies were included in the pooled analysis. The limited number of the included studies might be a possible explanation of the non-significant association between rs8179183 (Lys656Asn) and the CVD risk. Further study is still needed to confirm the association in different populations. Second, considerable heterogeneity was revealed across the studies. Differences of race and CVD disease type (heart failure, coronary heart disease, myocardial infarction, and stroke) may partially explain the significant heterogeneity. In addition, various adjusted confounders, different study designs, and other undetected factors may also lead to the presence of heterogeneity. Third, none of the included studies separately analyzed the relations among female and male participants. The gender-difference of the association might have been given and should be considered in the future. Fourth, the study population was relatively small except for the study by Elliott et al. [19]. This might lead to the probability of false-negative or false-positive results. Finally, a causal relationship between the LEPR gene variants (rs6700896, 109G allele, and 109GG genotype) and CVD risk cannot be established because of the observational nature of the present analysis. Therefore, the findings of our study should be cautiously interpreted.

## 5. Conclusions

In summary, our systematic review and meta-analysis indicated that the LEPR gene variants (rs6700896, 109G allele, and 109GG genotype) were significantly associated with the increased risk of CVD. However, the present study is limited in its small number of included studies, considerable heterogeneity, and observational study design. Further studies are still warranted to confirm the magnitude of the association.

## Figures and Tables

**Table 1:** Characteristics of the seven eligible studies.

First Author, Published Year	Ethnicity	Study Design	Male (%)	Mean Age (Years)	Case		Controls		Leptin Receptor Polymorphism
					Disease	No.	Matching	No.	
Abd El-Aziz, 2012 [10]	Egyptian	Case-control	26.0	55.5	Coronary artery disease	100	Age- and sex-matched healthy subjects	100	rs1137101 (Gln223Arg)
Aijälä, 2014 [18]	European	Cohort	49.1	51	Coronary artery disease, Cardiovascular disease	303,423	-	725,605	rs1137101 (Gln223Arg), rs1137100 (Lys109Arg)
Bienertová-Vašku, 2009 [11]	American	Case-control	71.2	56	Chronic heart failure	372	Age- and sex-matched healthy subjects	407	rs1137101 (Gln223Arg)
Elliott, 2009 [19]	European and Asian ^a^	Cohort	Both men and women, the proportion not reported	20–75 ^b^	Coronary heart disease	12,148	-	29,127	rs6700896
Jin, 2015 [20]	Asian	Case-control	53.3	59	Coronary artery disease	120	Age- and sex-matched healthy subjects	109	rs6700896
Roszkowska-Gancarz, 2014 [21]	European	Case-control	57.0	47	Myocardial infarction	226	Blood donors and volunteers	190	rs1137101 (Gln223Arg), rs1137100 (Lys109Arg), rs8179183 (Lys656Asn)
Tang, 2015 [12]	Asian	Case-control	67.5	61	Ischemic stroke	101	Age- and sex-matched healthy subjects	105	rs1137101 (Gln223Arg), rs1137100 (Lys109Arg), rs8179183 (Lys656Asn)

^a^ Data from the London Life Sciences Population study (LOLIPOP), the 1966 Northern Finnish Birth Cohort, the Lausanne Cohort (CoLaus), the Genetic Epidemiology of Metabolic Syndrome study (GEMS), the Epidemiological Study on the Insulin Resistance syndrome study (DESIR), and the LOLIPOP participants. ^b^ Min-max.

## Supplementary Materials

The following are available online at www.mdpi.com/1660-4601/14/4/375/s1, Table S1: Search strategy, Table S2: Quality assessment of the eligible studies (maximum score: 17), Table S3: Stratified analysis of the association between leptin receptor gene polymorphism and the risk of cardiovascular disease., Figure S1: Funnel plot for the analysis of the association between leptin receptor gene polymorphism and the risk of cardiovascular disease under three models: (a) Allelic, (b) Homozygous, and (c) Dominant.
