# Supplementary material for: Leptin Receptor Gene Polymorphism and the Risk of Cardiovascular Disease: A Systemic Review and Meta-Analysis
Source: Int J Environ Res Public Health. 2017 Apr 3;14(4):375. doi: 10.3390/ijerph14040375 (PMC5409576; doi:10.3390/ijerph14040375)
Supplement: Supplementary file 1 [file ijerph-14-00375-s001.pdf]

Supplementary Materials: Leptin Receptor Gene Polymorphism and the Risk of Cardiovascular Disease: A Systemic Review and Meta-Analysis

Lei Wu * and Dali Sun

Table S1. Search strategy. Source: PubMed (Searched on: October 9th, 2016).

#1 Leptin receptor[Title/Abstract]
#2 LEPR[title/abstract]
#3 OB-R[title/abstract]
#4 OB receptor[title/abstract]
#5 OBR[title/abstract]
#6 acute coronary syndromes
#7 myocardial infarction
#8 coronary artery disease
#9 coronary heart disease
#10 coronary disease
#11 ischemic heart disease
#12 cardiovascular disease
#13 stroke
#14 angina
#15 heart attack
#16 cerebrovascular*
#17 polymorphism
#18 Variant
#19 Variation
#20 genotype
#21 #1 OR #2 OR #3 OR #4 OR #5
#22 #6 OR #7 OR #8 OR #9 OR #10 OR #11 OR #12 OR #13 OR #14 OR #15 OR #16
#23 #17 OR #18 OR #19 OR #20
#24 #21 AND #22 AND #23

Source: Embase (October 9th, 2016)
#1 Leptin receptor:ti,ab
#2 LEPR:ti,ab
#3 OB-R:ti,ab
#4 OB receptor:ti,ab
#5 OBR:ti,ab
#6 acute coronary syndromes
#7 myocardial infarction
#8 coronary artery disease
#9 coronary heart disease
#10 coronary disease
#11 ischemic heart disease
#12 cardiovascular disease
#13 stroke
#14 angina
#15 heart attack
#16 cerebrovascular
#17 polymorphism
#18 variant
#19 variation
#20 genotype
#21 #1 OR #2 OR #3 OR #4 OR #5
#22 #6 OR #7 OR #8 OR #9 OR #10 OR #11 OR #12 OR #13 OR #14 OR #15 OR #16
#23 #17 OR #18 OR #19 OR #20
#24 #21 AND #22 AND #23

Table S2. Quality assessment of the eligible studies (maximum score: 17).

First author, published year	Design bias	Selection bias	Information bias	Confounding	Analysis bias	Total
Abd El-Aziz, 2012	1	4	5	2	2	14
Aijälä, 2014	3	3	5	3	2	16
Bienertová-Vašku°, 2009	1	4	5	3	2	15
Elliott, 2009	3	4	5	3	2	17
Jin, 2015	1	4	5	2	2	14
Roszkowska-Gancarz, 2014	1	3	5	0	2	11
Tang, 2015	1	4	5	2	2	14

Selection column includes five items: (1) Design bias: study design and follow up; (2) Selection bias: inclusion/exclusion criteria, recruitment strategy, interval between exposure and outcome assessment, and attrition; (3) Information bias: pre-specified outcome, outcome assessment, reliability of the outcome, data collection and assessment, and exposure assessment; (4) Confounding: confounding adjustment; and (5) Analysis bias: effect size and data availability.

Table S3. Stratified analysis of the association between leptin receptor gene polymorphism and the risk of cardiovascular disease.

Outcome	Allelic		Homozygous		Dominant	
	Comparisons, No.	OR (95% CI)	Comparisons, No.	OR (95% CI)	Comparisons, No.	OR (95% CI)
Total	14	1.10 (0.99, 1.22)	13	1.18 (0.89, 1.57)	13	1.11 (0.91, 1.36)
Ethnicity						
Caucasian	9	1.05 (0.95, 1.17)	9	1.19 (0.90, 1.57)	9	1.12 (0.92, 1.36)
Asian	4	1.71 (0.90, 3.26)	4	1.74 (0.37, 8.23)	4	1.28 (0.42, 3.85)
Mixed	1	1.06 (1.02, 1.10)	-	-	-	-
P-value for difference		0.407		0.936		0.799
Study design						
Case-control	9	1.31 (0.89, 1.43)	9	1.05 (0.68, 1.61)	9	0.95 (0.75, 1.21)

Cohort	5	1.07 (1.03, 1.11)	4	1.35 (0.93, 1.96)	4	1.39 (1.08, 1.79)
P-value for difference		0.991		0.443		0.078
Disease type						
Heart disease	11	1.06 (0.99, 1.13)	10	1.13 (0.85, 1.50)	10	1.09 (0.89, 1.33)
Stroke	3	2.42 (1.60, 3.65)	3	3.97 (0.90, 17.49)	3	1.92 (0.68, 5.40)
P-value for difference		0.003		0.185		0.486

OR, odd ratio; CI, confidence interval

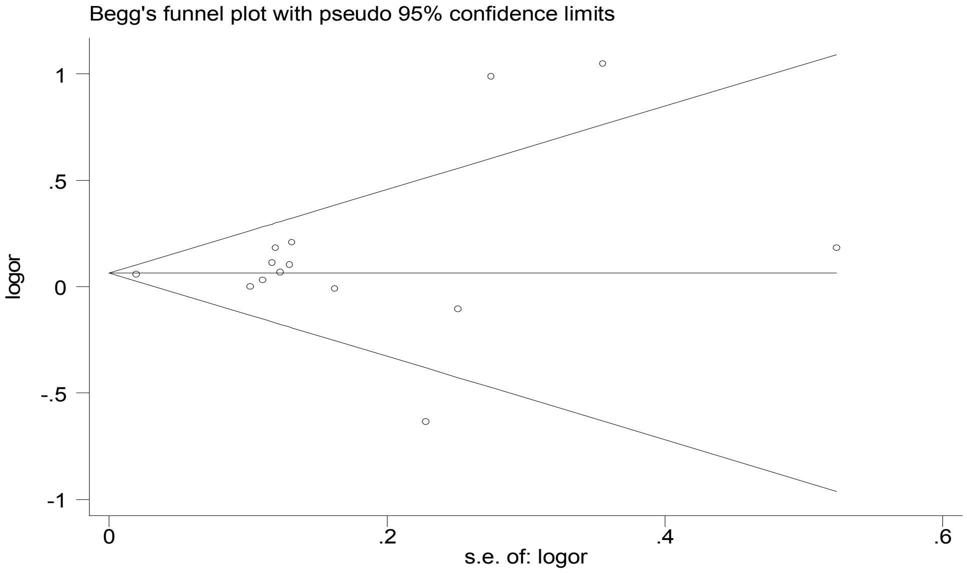

(a)

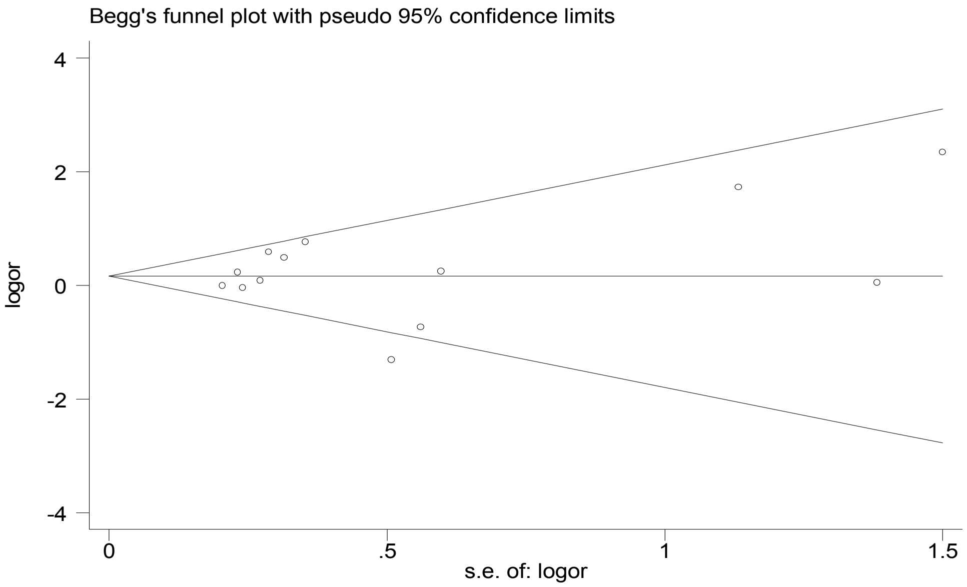
Begg's funnel plot with pseudo 95% confidence limits
4
2
0
-2
-4
logor
0
.5
1
1.5
s.e. of: logor

(**b**)

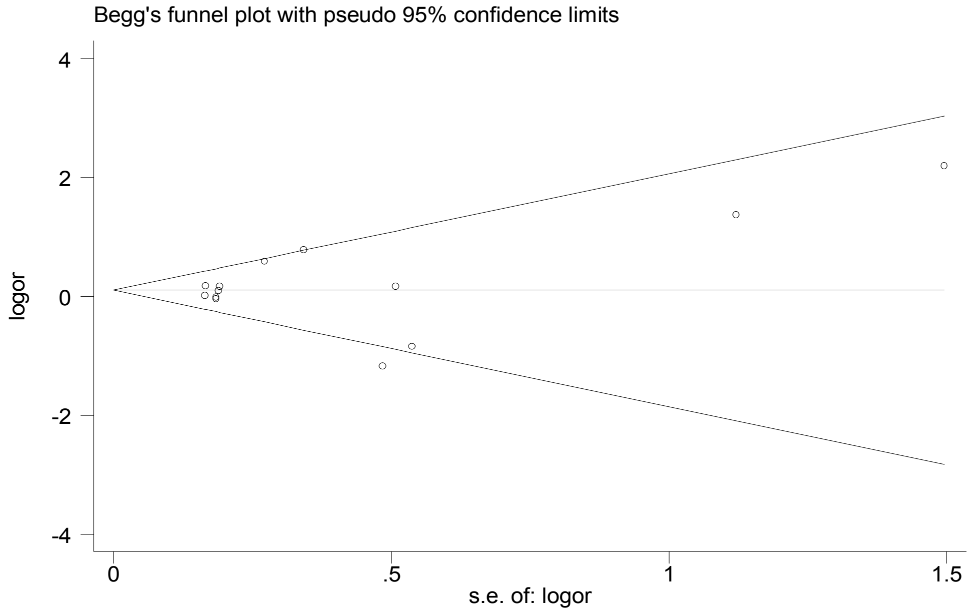

(**c**)

Figure S1. Funnel plot for the analysis of the association between leptin receptor gene polymorphism and the risk of cardiovascular disease under three models: (a) Allelic, (b) Homozygous, and (c) Dominant.

© 2017 by the authors; licensee MDPI, Basel, Switzerland. This article is an open access article distributed under the terms and conditions of the Creative Commons by Attribution (CC-BY) license (http://creativecommons.org/licenses/by/4.0/).
